# A Highly Efficient Synthesis of Polyubiquitin Chains

**DOI:** 10.1002/advs.201800234

**Published:** 2018-05-14

**Authors:** Qian Qu, Man Pan, Shuai Gao, Qing‐Yun Zheng, Yuan‐Yuan Yu, Jia‐Can Su, Xiang Li, Hong‐Gang Hu

**Affiliations:** ^1^ School of Pharmacy Second Military Medical University 325 Guohe Road Shanghai 200433 China; ^2^ Tsinghua‐Peking Center for Life Sciences Tsinghua University Beijing 100084 China; ^3^ Changhai Hospital Second Military Medical University 168 Changhai Road Shanghai 200433 China

**Keywords:** high‐efficiency solid phase peptide synthesis (HESPPS), microwave‐assisted synthesis, peptidesynthesis, polyubiquitin

## Abstract

A robust, microwave‐assisted, highly efficient, solid‐phase peptide synthesis method for preparing isopeptide‐linked 62‐mer and 76‐mer isoubiquitins and polyubiquitin is developed. The strategy avoids the use of costly resins and pseudoprolines, and the isopeptide‐linked building blocks can be assembled with high initial purity within 1 day. All seven diubiquitins are successfully synthesized on a multi‐milligram scale; a four‐segment, three‐ligation method is used to obtain a K33‐/K11‐linked mixed triubiquitin in excellent yield. Circular dichroism and crystallographic analyses are used to verify the structures of the well‐folded, synthetic polyubiquitin chains. The facile synthetic strategy is expected to be generally applicable for the rapid synthesis of isopeptide‐linked isoUbs and to pave the way for the study of longer polyubiquitin chains.

## 1 Introduction

Ubiquitylation is one of the most important post‐translational modifications (PTMs), and it is involved in a wide range of biological pathways including protein degradation, DNA repair, and cell‐cycle regulation.^1^, ^2^, ^3^, ^4^, ^5^ The process of ubiquitylation involves three main steps: activation, conjugation, and ligation, which are performed by ubiquitin‐activating enzymes (E1), ubiquitin‐conjugating enzymes (E2), and ubiquitin ligases (E3), respectively.^6^, ^7^, ^8^, ^9^, ^10^, ^11^ The result of this sequential cascade is the conjugation of the C‐terminal of the 76‐amino‐acid‐long ubiquitin (Ub) with the ε‐amine of lysine (Lys, K) in the substrate protein via an isopeptide bond. Notably, after conjugation with the substrate protein, a Lys in Ub can accept another ubiquitin molecule, giving rise to polyubiquitination of the substrate protein. Seven lysines in Ub (Lys6, Lys11, Lys27, Lys29, Lys33, Lys48, and Lys63) can be linked to the C‐terminal of Ub molecules in this way, and the resultant Ub chains can be either homogeneous (modified on the same Lys residue) or heterogeneous (modified on different Lys residues).^12^, ^13^, ^14^, ^15^ The exact nature of the polyubiquitination determines the fate of the substrate protein,^16^, ^17^, ^18^, ^19^ and therefore the functional decoding of polyUb chains is of great interest in biochemistry and structural biology.^20^, ^21^, ^22^, ^23^, ^24^

The development of an efficient, scalable, inexpensive synthesis of high‐purity, link‐defined polyUb chain is essential for biochemical and structural studies of polyUb chains,^25^, ^26^, ^27^ and both enzymatic and chemical syntheses of polyUb chains are known. In vitro Ub enzyme systems were first developed for the preparation of polyUbs, and their utility has been demonstrated by the synthesis of almost all types of Ub chains.^15^, ^28^, ^29^, ^30^, ^31^ However, K27‐polyUbs and heterogeneous polyUbs have yet to be accessed with this technology.^32^ Protein chemical synthesis has been recognized as a powerful means of obtaining well‐defined polyUb chains, and since the first chemical ubiquitination through a N‐α‐auxiliary‐mediated ligation was reported in 2007,^33^ many attempts have been made to improve the synthetic yield and develop novel strategies for the synthesis of the ubiquitinated proteins and polyUb chains.^33^, ^34^, ^35^, ^36^, ^37^, ^38^, ^39^, ^40^, ^41^, ^42^, ^43^, ^44^, ^45^, ^46^, ^47^, ^48^, ^49^, ^50^ For example, Brik and co‐workers and Ovaa and co‐workers realized the chemical synthesis of polyUb chains through γ‐ or δ‐thiolysine‐mediated chemical ligations;^34^, ^35^, ^36^, ^37^ Chatterjee and co‐workers used a 2‐aminooxy ethanethiol auxiliary to mediate chemical ubiquitination;^38^ and a genetically encoded, orthogonal protection and activated ligation (GOPAL) approach also proved to be efficient.^39^

Similar to the aforementioned strategies, diUb is usually synthesized by the assembly and subsequent N‐α‐auxiliary‐mediated ligation from monoUb – a cumbersome, expensive, and low‐yielding procedure that greatly restricts access to the large quantities of highly pure and well‐folded polyUbs necessary for further biological studies. Recently, Tang et al.^51^ reported a new isoUb‐based strategy using a readily accessible, premade, isopeptide‐linked 76‐mer isoUb, which avoids the use of an auxiliary‐modified Lys. Aided by unconventional resin and pseudoproline, they successfully obtained isopeptide‐linked 76‐mer isoUb using regular solid‐phase peptide synthesis (SPPS) techniques. However, the reported method has three limitations: 1) the ChemMatrix resin used, though commercially available, is prohibitively expensive; 2) the incorporation of two pseudoproline building blocks during SPPS increases the number of reaction steps, lowering the synthetic yield; and 3) the regular SPPS process used to produce the isoUb is quite time‐consuming.^51^

**Figure 1:**
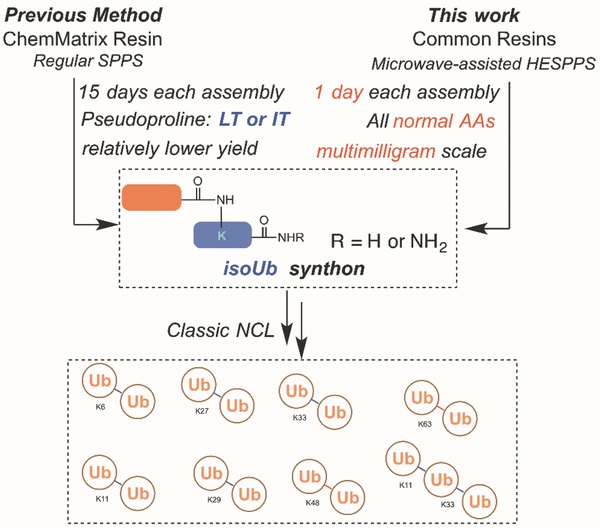
Chemical synthesis of polyUbs. Previous method: Regular SPPS for isoUb‐mediated native chemical ligation. This work: HESPPS for isoUb‐mediated native chemical ligation. HESPPS: high‐efficiency solid‐phase peptide synthesis; Ub: ubiquitin.

Accordingly, we sought an efficient, economical, and convenient strategy for preparing polyUb chains. Microwave irradiation has been widely used in SPPS and offers significant improvements in terms of efficiency, speed, and reduced chemical waste, especially for long peptides, in comparison with conventional SPPS.^52^, ^53^, ^54^, ^55^, ^56^, ^57^, ^58^, ^59^ Herein, we disclose the microwave‐assisted, high‐efficiency solid phase peptide synthesis (HESPPS) of these key isopeptide‐containing isoUbs with good initial purity and high efficiency, which allows the facile synthesis of polyUbs (**Figure** **1**).

## 2 Results and Discussion

### 2.1 HESPPS‐Enabled Preparation of Ub Segments

**Figure 2:**
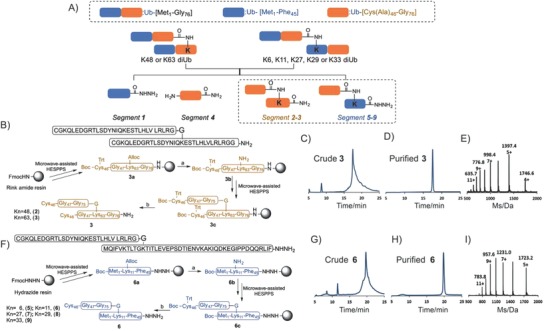
Synthesis of the isoUb building blocks: A) Retrosynthetic analysis of diUbs; B) synthesis of key isoUb segment **3**; C) analytical HPLC chromatogram of crude product **3**; D) analytical HPLC chromatogram of isolated product **3**; E) observed ESI‐MS spectrum of the main peak of **3**; F) synthesis of key isoUb segment **6**; G) analytical HPLC chromatogram of crude product **6**; H) analytical HPLC chromatogram of isolated product **6**; and I) observed ESI‐MS spectrum of the main peak of **6**. Reagents and conditions: a) Pd[P(Ph)_3_]_4_, phenylsilane/DCM; b) TFA/phenol/water/thioanisole/1,2‐ethanedithiol (82.5/5/5/5/2.5, v/v). HPLC (λ = 214 nm).

Ala46 in each Ub unit was temporarily mutated to Cys for the ligation, and thus all seven diUbs can be classified in two categories based on the location of the isopeptide bond with the first category consisting of K48‐ and K63‐diUb and the second consisting of K6‐, K11‐, K27‐, K29‐, and K33‐diUb. For the synthesis of K48‐ or K63‐diUb, a 45‐mer Ub[Met_1_—Phe_45_]—NHNH_2_ (**1**) and 62‐mer Ub[Cys_46_—(Cys_46_—Gly_76_)Lys*_n_*—Gly_76_]—NH_2_ (**2** and **3**, *n* = 48 for **2** and *n* = 63 for **3**) need to be assembled in the first step. For K6‐, K11‐, K27‐, K29‐, or K33‐diUb, the necessary segments are a 45‐mer Ub[Met_1_—Phe_45_]—NHNH_2_ (**1**), 31‐mer Ub[Cys_46_—Gly_76_]—NH_2_ (**4**), and 76‐mer Ub[Met_1_—(Cys_46_—Gly_76_)Lys*_n_*—Phe_45_]—NHNH_2_ (**5**–**9**, *n* = 6 for **5**, *n* = 11 for **6**, *n* = 27 for **7**, *n* = 29 for **8**, and *n* = 33 for **9**) (**Figure** **2**A). Herein lies the major challenge of our design; the efficient preparation of high‐purity samples of segments **2**, **3** and segments **5**–**9** (the synthesis of pure, crystalline samples of segments **1** and **4** has been previously accomplished^51^ using a Fmoc‐(9‐fluorenylmethoxycarbonyl)‐based approach at 75 °C (Figure S1, Supporting Information)).

First, fragment **3a** was prepared using a standard HESPPS procedure, with Rink amide resin as the solid support, and Fmoc‐Lys(Alloc)—OH was installed at the linkage site (Alloc, allyloxycarbonyl). Then, the Alloc group was removed using Pd[P(Ph)_3_]_4_ and phenylsilane to yield fragment **3b** on the resin.^60^, ^61^, ^62^, ^63^ Next, another equivalent of Ub—[Cys_46_—Gly_76_]—OH was attached to the proximal Lys ε‐amine of the linkage site. Finally, the resin was treated with reagent K (trifluoroacetic acid (Tfa)/H_2_O/thioanisole/1,2‐ethanedithiol (EDT) 87.5/5/5/2.5, v/v/v/v) to release the crude peptide. Unfortunately, under standard 75 °C coupling conditions, no apparent main peak was observed in the analytical trace of crude **3**, indicating pure **3** had not been generated. Accordingly, the coupling was attempted at 90 °C instead (Figure 2B), which provided segment **3** with good initial purity (Figure 2C). Purification by preparative reversed‐phase high‐performance liquid chromatography (RP‐HPLC) yielded highly pure **3**, as verified by electrospray ionization mass spectrometry (ESI‐MS) (Figure 2D,E). Using this method, 142 mg of segment **3** could be synthesized, purified, and lyophilized in just 4 days starting from only 500 µmol of Rink amide resin (overall 4.1% yield and 98% purity). Applying the same strategy, segment **2** was obtained in high purity (98%) and on a similar scale to what was achieved with **3** (196 mg with overall 5.7% yield) (Figure S2, Supporting Information).

Our approach to segment **6** is depicted in Figure 2F. First, fragment **6a** was synthesized by a standard HESPPS procedure with 2‐chlorotrityl‐based hydrazine resin as the solid support, and the orthogonal Alloc‐protected group was selectively introduced at the K11 ε‐amine. The Alloc group was then removed to give **6b**, which was elongated with the Ub sequence (Cys46—Gly76) to yield **6c**. Finally, standard cleavage released crude peptide **6** with good initial purity (Figure 2G), which was purified by preparative RP‐HPLC to give a highly pure sample of **6**, as verified by ESI‐MS (Figure 2H,I). Using this method, 181 mg of segment **6** could be synthesized, purified, and lyophilized in just 4 days starting from 500 µmol of hydrazide resin (overall 4.2% yield and 98% purity). An analogous strategy was used to synthesize segments **5** and **7**–**9** in high purity (98%) and on a similar scale (overall yields from 3.6% to 5.1%) (Figures S3–S6, Supporting Information).

### 2.2 Preparation of diUb by Hydrazide‐Based Native Chemical Ligation

**Figure 3:**
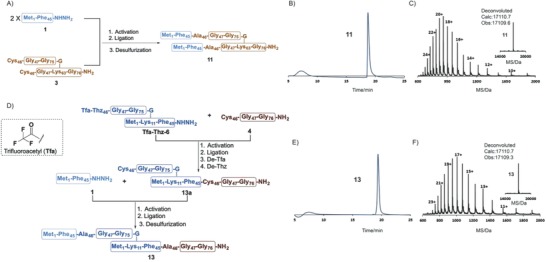
Synthesis of K63‐diUb **10**: A) Overall synthetic route; B) analytical HPLC chromatogram of purified **10**; C) observed ESI‐MS spectrum of purified **11** (24+, 713.9; 22+, 778.7; 20+, 856.5; 18+, 951.5; 16+, 1070.4; 14+, 1223.1; 12+, 1426.8; and 10+, 1712.0); inset: deconvoluted ESI‐MS spectrum of **11**, observed mass = 17 109.6 Da, calculated mass = 17 110.7 Da, average isotopes). Synthesis of K11‐diUb **13**: D) Overall synthetic route; E) analytical HPLC chromatogram of purified **13**; F) observed ESI‐MS spectrum of purified **13** (23+, 744.9; 21+, 815.7; 19+, 901.5; 17+, 1007.4; 15+, 1141.6; 13+, 1317.1; and 11+, 1556.4); inset: deconvoluted ESI‐MS spectrum of **13** (observed mass = 17 109.3 Da, calculated mass = 17 110.7 Da, average isotopes). Reactions and conditions: activation: 6 m Gn‐HCl, 10 equiv. NaNO_2_, pH 3.0; ligation: 6 m Gn‐HCl, 40 equiv. MPAA, pH: 6.4; desulfurization: 6 m Gn‐HCl, 500 × 10^−3^ m TCEP, tBuSH, VA‐044, pH: 6.9; de‐Tfa: 2 m NaOH, pH: 10.0; de‐Thz: methoxyamine‐HCl, pH = 4.0. HPLC (λ = 214 nm).

With key segments **1**–**9** in hand, our attention turned to the synthesis of each diUb. For the synthesis of K48‐diUb **10** and K63‐diUb **11**, two equivalents of segment **1** were ligated with one equivalent of either segment **2** (for K48) or **3** (for K63) in a single hydrazide‐based native chemical ligation^64^, ^65^, ^66^, ^67^, ^68^, ^69^, ^70^ step followed by a single desulfurization procedure.^42^, ^71^, ^72^ One representative ligation process between **1** and **3** was complete within 12 h, and the subsequent desulfurization step afforded full‐length K63‐diUb **11** on an 80 mg scale in an overall yield of 32%, as confirmed by HPLC and ESI‐MS (**Figure** **3**A–C and Figure S7 (Supporting Information)). K48‐diUb **10** was also successfully prepared using the same method (75 mg scale in an overall 30% yield) (Figure S2D, Supporting Information).

We used a one‐pot ligation strategy to further simplify the syntheses of K6‐, K11‐, K27‐, K29‐, and K33‐diUbs, compounds **12**–**16**, respectively, all of which were synthesized in overall yields ranging from 14% to 22% (Figures S3–S6, Supporting Information). As an example, K11‐diUb **13** was synthesized by hydrazide‐based native chemical ligation^64^, ^65^, ^66^, ^67^, ^68^, ^69^, ^70^ of segment **4** with 76‐mer Ub[Met_1_—(Tfa—Thz_46_—Gly_76_)Lys_11_—Phe_45_]—NHNH_2_ (the Cys46 of **6** was mutated to Tfa—Thz, and the compound was named **Tfa‐Thz‐6**) followed by Tfa—Thz deprotection to give **13a**, which was ligated with segment **1**. After desulfurization, the final product, K11‐diUb **13**, was obtained in an overall yield of 20% on a 50 mg scale (Figure 3D–F).

### 2.3 Structural Characterization of Synthetic diUbs Prepared by Coupling at 90 °C

**Figure 4:**
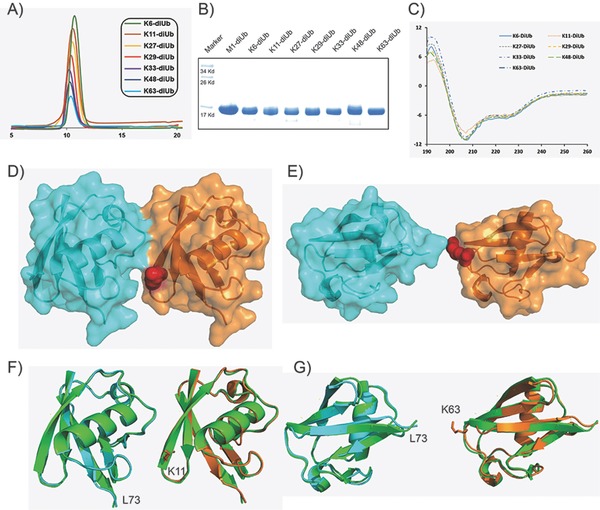
Characterization of diUbs: A) FPLC chromatogram; B) SDS‐PAGE; C) circular dichroism spectra; crystal structures of D) **K11‐diUb** and E) **K63‐diUb** with K11 and K63 shown as red balls; structural alignment of wild‐type Ub with F) **K11‐diUb** units; and G) **K63‐diUb** units with the wild‐type Ubs shown as green cartoons.

We sought to determine whether the synthetic diUbs prepared at relatively high coupling temperatures can be folded into the proper conformation. First, samples of all the synthetic diUbs were folded by the urea‐gradient dialysis method and then characterized by fast protein liquid chromatography (FPLC) and sodium dodecyl sulfate polyacrylamide gel electrophoresis (SDS‐PAGE). All the folded Ubs exhibited excellent homogeneity and purity (**Figure** **4**A,B), and the circular dichroism (CD) spectra of the correctly folded diUbs, which exhibited negative peaks at 208 and 226 nm, were consistent with those of wild‐type Ub (Figure 4C).^73^

Diffraction‐quality crystals of homogeneous K11‐diUb and K63‐diUb were successfully obtained by monomer/oligomer racemic crystallization,^42^ solved by molecular replacement, and refined to give the final statistics (Table S2, Supporting Information). K11‐diUb was found to adopt a compact conformation, which is consistent with previous studies (Figure 4D),^31^ whereas K63‐diUb took on an open conformation (Figure 4E). The structural alignment of wild‐type Ub with the Ub units in K11‐ and K63‐diUb very strongly suggests that all the Ub units adopted the native conformation (Figure 4F,G). Taken together, these results show that the coupling conditions used result not only in high‐purity samples of the Ub chains but in samples that are spectroscopically indistinguishable from the wild type.

### 2.4 HESPPS‐Enabled the Synthesis of K33‐/K11‐Linked Mixed Ub Chains

**Figure 5:**
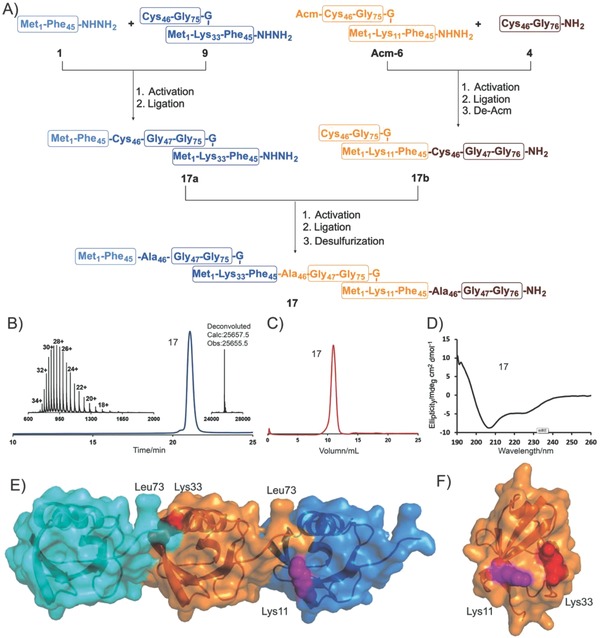
Synthesis and characterization of K33‐/K11‐linked Mixed Ub chain **17**: A) Overall synthetic route; B) analytical HPLC chromatogram and observed ESI‐MS spectrum of purified **17** (34+, 755.6 Da; 32+, 802.8 Da; 30+, 856.3 Da; 28+, 917.4 Da; 26+, 987.8 Da; 24+, 1070.1 Da; 22+, 1167.2 Da; 20+, 1283.9 Da; and 18+, 1426.4 Da); inset: deconvoluted of ESI‐MS spectrum of **17** (observed mass = 25 655.5 Da, calculated mass = 25 657.5 Da, average isotopes); C) FPLC chromatogram; D) CD spectra; E) crystal structure of K33‐/K11‐linked triUb **17**; F) Ub molecule within one asymmetric unit; Lys11 is shown as a purple ball, and Lys33 is shown as a red ball. Reactions and conditions: activation: 6 m Gn‐HCl, 10 equiv. NaNO_2_, pH 3.0; ligation: 6 m Gn‐HCl, 40 equiv. MPAA, pH: 6.4; de‐Acm: 60 equiv. PdCl_2_, pH 6.4; desulfurization: 6 m Gn‐HCl, 500 × 10^−3^ m TCEP, tBuSH, VA‐044, pH: 6.9. HPLC (λ = 214 nm).

Next, we applied our new strategy to the assembly of the 228‐mer K33‐/K11‐linked mixed triUb chain, which is involved in immune response, cell‐cycle progression, and protein trafficking.^74^ As depicted in **Figure** **5**A, four segments, namely, **1**, **9**, **Acm‐6** (with Cys46 of **6** protected by an acetamidomethyl (Acm) group) and **4**, must be synthesized and assembled. With the aid of HESPPS, these four segments were all obtained on a hundred‐milligram scale. First, ligation between segments **1** and **9** afforded **17a**. In addition, ligation between segments **Acm‐6** and **4** followed by cleavage of the Acm protecting group^75^ was conducted in one pot to produce **17b**. Finally, intermediate segments **17a** and **17b** were ligated to generate the full‐length triUb, which was subjected to desulfurization to convert Cys46 in all three Ub units into Ala46. The native K33‐/K11‐linked mixed triUb **17** was obtained in an overall yield of 8.0% on a 20 mg scale, and the materials were characterized by HPLC and ESI‐MS (Figure 5B and Figure S10 (Supporting Information)). After folding by urea‐gradient dialysis, size exclusion chromatography showed that the synthesized sample of correctly folded **17** exhibited excellent homogeneity (Figure 5C). CD spectroscopy showed that the conformation of **17** was consistent with that of monoUb (Figure 5D). The presence of two negative peaks at 208 and 226 nm suggested that each Ub unit in the synthetic triUb chain retained its globular conformation.^42^ Diffraction‐quality crystals of **17** were obtained by monomer/oligomer racemic crystallization (Figure 5E); the structure was solved by molecular replacement and was refined to give the final statistics.^42^

We first solved the crystal structure of the K33‐/K11‐linked mixed triUb, which was found to contain only one Ub molecule per asymmetric unit. Although the C‐terminal was poorly ordered (as previously reported^38^), the combination of adjacent asymmetric units shows two possible patterns for the formation of the isopeptide bond. With a more detailed perspective, the residues of K11 and K33 were found to be in similar orientations, indicative of the complimentary roles of these two lysines in chain formation (Figure 5F).

## 3 Conclusions

A HESPPS–based strategy for the rapid chemical preparation of Ub chains has been developed. Segments bearing isopeptide bonds can be directly prepared on normal resins (Rink amide resin and 2‐chlorotrityl resin) and assembled within 1 day without requiring any pseudoprolines. The utility of this new strategy was exemplified in the synthesis of all seven types of diUbs in high purities and excellent yields on 10–100 mg scales. Crystallographic analyses of K11‐diUb and K63‐diUb confirmed that under the high reaction temperatures (90 °C) used during the HESPPS process, the synthetic Ubs can be folded in the correct conformations. To further showcase our new strategy, K33‐/K11‐linked mixed triUb chains were successfully synthesized on a tens of milligrams scale, and their crystal structures were resolved for the first time. Interestingly, the K11‐linked Ub motif adopts the same orientation as the K33‐linked Ub motif, suggesting that K11 and K33 may play complementary roles in the formation of Ub chains. Taken together, these results prove our strategy to be a vast improvement over previous approaches to the preparation of polyUb chains, and it should be capable of delivering sufficient quantities of high‐purity polyUb chains for biological and structural research.

## 4 Experimental Section

*General Fmoc Solid‐Phase Peptide Synthesis*: All the peptides were synthesized using standard Fmoc SPPS protocols under microwave conditions (CEM Liberty Blue). Resins (Rink amide AM resin or Fmoc‐hydrazine 2‐chlorotrityl chloride resin) were selected based on the peptide segments. Resins were swelled in *N*,*N*‐dimethylformamide (DMF) for 10 min prior to use. Each amino acid was coupled under different conditions during the coupling cycle. In general, the first 30 amino acids in the amino acid sequence were coupled using the microwave method for standard single coupling (Table S1A, Supporting Information) or for His and Cys, a standard single coupling method at 50 °C (Table S1C, Supporting Information). Subsequent amino acids in the amino acid sequence were coupled using the microwave method for standard double coupling (Table S1B, Supporting Information) or a standard double coupling method at 50 °C (Table S1D, Supporting Information). Each standard coupling cycle includes Fmoc deprotection using 20% piperidine in DMF and amino acid coupling using a fourfold excess of 0.2 m Fmoc‐protected amino acid in DMF, 1.0 m *N*,*N′*‐diisopropylcarbodiimide (DIC) in DMF, and 1.0 m Oxyma in DMF. Fmoc‐Lys(Alloc)—COOH was introduced for the construction of the isopeptide bonds. Alloc (0.25 mmol) was removed using Pd[P(Ph)_3_]_4_ (60 mg) and phenylsilane (600 µL) in 5 mL of dichloromethane (DCM) for 3 h. The peptide chain elongation continued until the last amino acid deprotection. After the completion of the SPPS, the peptide–resin was transferred into a customized sand core funnel and treated with reagent K for 2 h at room temperature. Crude peptides were precipitated with cold diethyl ether and then dissolved in water (containing 0.1% Tfa) mixed with acetonitrile (containing 0.1% Tfa) for HPLC analysis and purification.

*Hydrazide‐Based Native Chemical Ligation*: The hydrazide peptide (1 µmol, 1 equiv.) was dissolved in 1 mL of cold (−20 °C) ligation buffer (6 m Gn‐HCl, 100 × 10^−3^ m NaH_2_PO_4_, pH 3.0). NaNO_2_ (10 equiv.) was added to the reaction buffer at −10 to −20 °C. The reaction was incubated for 30 min at −10 °C to fully convert the hydrazide to the acyl azide. 4‐Mercaptophenylacetic acid (MPAA) (40 equiv.) was added to the reaction buffer, and the pH of the solution was adjusted to 5.0 with 2 m aqueous NaOH. Finally, the N‐terminal Cys peptide (1 µmol, 1 equiv.) was added, and the pH of the solution was adjusted to 6.5 to initiate the ligation. The reaction was monitored by analytical RP‐HPLC.

*Desulfurization Reaction*: Peptides were dissolved in desulfurization buffer (6 m Gn‐HCl, 0.1 m Na_2_HPO_4_, 500 × 10^−3^ m tris(2‐carboxyethyl)phosphine (TCEP), pH 6.9) to give a peptide concentration of 0.6 × 10^−3^ m. tert‐Butanethiol (tBuSH) (106 µL µmol^−1^ peptide) and VA‐044 (34 mg µmol^−1^ peptide) were added, and the mixture was stirred overnight at 37 °C. The reaction was monitored by analytical RP‐HPLC.

*Deprotection of Tfa–Thz*: The deprotection of Tfa–Thz to yield the free N‐terminal Cys derivative was performed in one pot. After the first ligation was completed, clean‐Tfa deprotection was performed at pH 10.0 for 30 min (partial Tfa deprotection was observed during the ligation process), then Thz was converted to Cys by adding methoxyamine (0.2 m) and allowing the mixture to react at pH 4.0 for 2 h.

*Acm Deprotection*: The one‐pot ligation and Acm deprotection reaction were performed as reported.^75^ First, 40 equiv. of MAPP was used in the hydrazide‐based ligation. After the ligation was complete, 10 equiv. of TCEP and 60 equiv. of PdCl_2_ were added, and the mixture was incubated for 0.5 h to cleave the Acm group. Finally, after the addition of dithiothreitol (DTT) (1 m), the product was purified using RP‐HPLC.

*Synthetic Protein Folding*: Lyophilized polyubiquitin chains were dissolved in buffer (100 × 10^−3^ m Na_2_HPO_4_, 8 m urea; pH 3.0) at a protein concentration of 20 mg mL^−1^ and then dialyzed to 2 mg mL^−1^ with the buffer solution (20 × 10^−3^ m Tris, 150 × 10^−3^ m NaCl, pH 7.5). The diluted protein solution was centrifuged at 14 000 rpm prior to loading onto a preequilibrated Superdex 75 column (GE Life Sciences) using an AKTA FPLC system. The flow rate of the elution buffer (50 × 10^−3^ m NaCl, 20 × 10^−3^ m Tris, pH 7.5) was 0.4 mL min^−1^. Fractions containing pure Ub chains were concentrated and frozen at −80 °C.

*CD Experiments*: Circular dichroism experiments were conducted at 298 K using an Applied Photophysics Pistar π‐180 CD spectrometer, and the experiments were conducted at the Center of Biomedical Analysis, Tsinghua University. The concentrations of K6‐diUb, K11‐diUb, K27‐diUb, K29‐diUb, K33‐diUb, K48‐diUb, K63‐diUb, and K33‐/K11‐triUb were 0.1–0.2 mg mL^−1^, and the width of the CD cuvette was 1 mm. The wavelength range was 190–260 nm, and the step was 1 nm. Each sample was analyzed three times.

*Synthesis of d‐Ub: d*‐monoUb was prepared as previously reported.^42^ A two‐segment hydrazide‐based native chemical ligation was used to prepare *d*‐monoUb in which the Ala46 was temporarily mutated to Cys46. Met1 was replaced with Nle to avoid the oxidation of Met during the synthesis. Both the segments were synthesized through HESPPS (Figure 1). After ligation, desulfurization was conducted to convert Cys46 to Ala46. The obtained *d*‐monoUb was characterized with HPLC, ESI‐MS, and CD (Figure S11, Supporting Information).

*X‐Ray Crystal Structure Determination*: The protein solution consisted of 1.5 mg mL^−1^ of both Ub chain samples and diUbs (3 mg mL^−1^ total protein) in 20 × 10^−3^ m Tris and 50 × 10^−3^ m NaCl buffer (pH 7.5). Crystals of a racemic mixture of K11‐diUb, K63‐diUb, and K33‐/K11‐triUb were grown in sitting drops at 293 K. Crystals grew overnight and reached their maximum size after two weeks. X‐ray diffraction data were collected on a Rigaku Micromax system. Structures were solved by molecular replacement and refined to give the final statistics (Table S2, Supporting Information).^42^

## Conflict of Interest

The authors declare no conflict of interest.

## Supporting information

Supplementary
