# Supplementary material for: A Highly Efficient Synthesis of Polyubiquitin Chains
Source: Adv Sci (Weinh). 2018 May 14;5(7):1800234. doi: 10.1002/advs.201800234 (PMC6051384; doi:10.1002/advs.201800234)
Supplement: Supplementary file 1 — Supplementary [file ADVS-5-1800234-s001.pdf]

Copyright WILEY-VCH Verlag GmbH & Co. KGaA, 69469 Weinheim, Germany, 2018.

ADVANCED SCIENCE
Open Access

Supporting Information

for *Adv. Sci.,* DOI: 10.1002/advs.201800234

A Highly Efficient Synthesis of Polyubiquitin Chains

Qian Qu, Man Pan, Shuai Gao, Qing-Yun Zheng, Yuan-Yuan Yu, Jia-Can Su, Xiang Li,* and Hong-Gang Hu**

Copyright WILEY-VCH Verlag GmbH & Co. KGaA, 69469 Weinheim, Germany, 2016.

Supporting Information

A Highly Efficient Synthesis of Polyubiquitin Chains

Qian Qu, Man Pan, Shuai Gao, Qing-Yun Zheng, Yuan-Yuan Yu, Jia-Can Su, Xiang Li,* and Hong-Gang Hu**

1. Materials and Methods

1.1 Materials

All reagents and solvents were purchased from J&K Scientific (Shanghai), GL Biotech (Shanghai), CSbio (Shanghai) or Sinopharm Chemical Reagent Co. Ltd and were used as supplied unless otherwise stated. Rink Amide MBHA resin and 2-chlorotrityl chloride resin were purchased from Tianjin Nankai Hecheng Science & Technology Co. Ltd. All the peptides were synthesized using standard Fmoc SPPS protocols under microwave conditions (CEM Liberty Blue).

1.2 HPLC and FPLC

Peptide fragments were analyzed by RP-HPLC using analytical columns (Grace Vydac C8, Welch XB-C18 and Welch XB-C4, 250 mm× 4.6 mm, 5 µm particle size, flow rate 1.0 mL/min) using Shimadzu instruments (Prominence LC-20AT) and purified by RP-HPLC using a semi-preparative column (Grace Vydac C8, 250× 25 mm, 10 µm particle size and Welch XB-C4, 150 mm× 21.2 mm and Welch XB-C18, 250 mm× 21.2 mm, 5 µm particle size, flow rate 7.0 mL/min) in Shimadzu instruments (Prominence LC-20AT). Analytical injections were monitored at 214 nm and 254 nm. The mobile phase was a mixture of acetonitrile (containing 0.1% TFA) and deionized, distilled water (containing 0.1% TFA). Both solvents were sonicated for 10 min before use. The purity of the product peptide was calculated based on the percent product peak area relative to the total peak area. A purity of 98% indicates the target peptide is at least 98% pure.

Refolded peptides were purified on a GE Healthcare (AKTA purifier 10 UPC-F920) instrument using a Superdex Peptide column, Superdex 75 column or Mono Q column. The eluate was monitored at 280 nm. All the buffers were sonicated for 10 min before use and filtered through 0.22-µm filter paper.

1.3 Mass spectrometry

Crude peptides and reaction products were characterized by normal ESI-MS on an LC/MS 2020 (Shimadzu) system. In addition, final products were characterized by high-resolution ESI-MS on a SYNAPTTM G2-Si HDMS system.

2. Tables and Figures

Table S1 The details of the microwave methods [a]

(A) Cycles for Standard Single Coupling

Standard Single Coupling		
	**Operation**	**Parameters**
**1**	**Deprotection**	Reaction Method: Standard Deprotection (**Table S1E**) Volume:7 mL
**2**	**Wash**	Volume 7 mL, Drain Time: 20 s
**3**	**Wash**	Volume 5 mL, Drain Time: 7 s
**4**	**Wash**	Volume 3 mL, Drain Time: 5 s
**5**	**Wash**	Volume 7 mL, Drain Time: 15 s
**6**	**Coupling**	Reaction Method: Standard Coupling (**Table S1F**) Amino Acid Volume: 5 mL; Activator Volume: 1.5 mL; Activator Base Volume: 1.5 mL; Manifold Wash Volume: 2 mL
**7**	**Wash**	Volume 5 mL, Drain Time: 10 s
**8**	**Wash**	Volume 3 mL, Drain Time: 5 s
**9**	**Wash**	Volume 7 mL, Drain Time: 15 s

(B) Cycles for Standard Double Coupling

Standard Double Coupling		
	**Operation**	**Parameters**
**1**	**Deprotection**	Reaction Method: Standard Deprotection (**Table S1E**) Volume:7 mL
**2**	**Wash**	Volume 7 mL, Drain Time: 20 s
**3**	**Wash**	Volume 5 mL, Drain Time: 7 s
**4**	**Wash**	Volume 3 mL, Drain Time: 5 s
**5**	**Wash**	Volume 7 mL, Drain Time: 15 s
**6**	**Coupling**	Reaction Method: Standard Coupling (**Table S1F**) Amino Acid Volume: 3.5 mL; Activator Volume: 1 mL; Activator Base Volume: 1 mL; Manifold Wash Volume: 2 mL
**7**	**Coupling**	Reaction Method: Standard Coupling (**Table S1F**) Amino Acid Volume: 3.5 mL; Activator Volume: 1 mL; Activator Base Volume: 1 mL; Manifold Wash Volume: 2 mL
**8**	**Wash**	Volume 5 mL, Drain Time: 10 s

**9**	**Wash**	Volume 3 mL, Drain Time: 5 s
**10**	**Wash**	Volume 7 mL, Drain Time: 15 s

(C) Cycles for Standard 50°C Single Coupling

Standard 50°C Single Coupling		
	**Operation**	**Parameters**
**1**	**Deprotection**	Reaction Method: Standard Deprotection (**Table S1E**)<br>Volume: 7 mL
**2**	**Wash**	Volume 7 mL, Drain Time: 20 s
**3**	**Wash**	Volume 5 mL, Drain Time: 7 s
**4**	**Wash**	Volume 3 mL, Drain Time: 5 s
**5**	**Wash**	Volume 7 mL, Drain Time: 15 s
**6**	**Coupling**	Reaction Method: 50°C Coupling (**Table S1G**)<br>Amino Acid Volume: 5 mL; Activator Volume: 1.5 mL; Activator Base Volume: 1.5 mL; Manifold Wash Volume: 2 mL
**7**	**Wash**	Volume 5 mL, Drain Time: 10 s
**8**	**Wash**	Volume 3 mL, Drain Time: 5 s
**9**	**Wash**	Volume 7 mL, Drain Time: 15 s

(D) Cycles for Standard 50°C Double Coupling

Standard 50°C Double Coupling		
	**Operation**	**Parameters**
**1**	**Deprotection**	Reaction Method: Standard Deprotection (**Table S1E**)<br>Volume: 7 mL
**2**	**Wash**	Volume 7 mL, Drain Time: 20 s
**3**	**Wash**	Volume 5 mL, Drain Time: 7 s
**4**	**Wash**	Volume 3 mL, Drain Time: 5 s
**5**	**Wash**	Volume 7 mL, Drain Time: 15 s
**6**	**Coupling**	Reaction Method: 50°C Coupling (**Table S1G**)<br>Amino Acid Volume: 3.5 mL; Activator Volume: 1 mL; Activator Base Volume: 1 mL; Manifold Wash Volume: 2 mL
**7**	**Coupling**	Reaction Method: 50°C Coupling (**Table S1G**)<br>Amino Acid Volume: 3.5 mL; Activator Volume: 1 mL; Activator Base Volume: 1 mL; Manifold Wash Volume: 2 mL
**8**	**Wash**	Volume 5 mL, Drain Time: 10 s
**9**	**Wash**	Volume 3 mL, Drain Time: 5 s

**10**	**Wash**	Volume 7 mL, Drain Time: 15 s

(E) Microwave Method for Standard Deprotection

Standard Deprotection		
**Temperature (°C)**	**Power (W)**	**Hold Time (s)**
**70**	160	15
**80**	30	75

(F) Microwave Method for Standard Coupling

Standard Coupling		
**Temperature (°C)**	**Power (W)**	**Hold Time (s)**
**75**	70	30
**85**	40	30
**89**	30	120

(G) Microwave method for 50°C Coupling

50ºC Coupling		
**Temperature (°C)**	**Power (W)**	**Hold Time (s)**
**30**	5	120
**50**	35	480

[a], Deprotection solvent: 20% piperidine/DMF; Activator: *N,N'*-diisopropylcarbodiimide (DIC) 0.5 M in DMF; Activator Base: Oxyma 1 M in DMF; Fmoc-Amino Acid: 0.2 M in DMF; and Manifold wash solvent: DMF.

Table S2. Data collection and structure refinement statistics

**Data collection**	**K11-diUb**	**K63-diUb**	**K33/11-triUb**
Spacegroup	P1	P1	P2
cell parameters	26.313 26.429 43.455<br>76.01 75.60 81.22	26.313 45.009 47.499<br>79.23 84.61 81.79	26.430 29.051 44.300<br>82.99 86.51 71.31
Resolution(Å)	2.09	2.34	1.81
Rmerge	0.058(0.422)	0.085(0.363)	0.042(0.138)
I /σI	16.03(2.45)	13.17(3.07)	26.56(8.23)
Completeness (%)	89.5(65.0)	94.8(93.9)	92.8(79.3)
Redundancy	2.8(2.3)	2.9(2.6)	3.3(2.9)
**Refinement**			
Resolution(Å)	2.091	2.344	1.812
No. reflections	5721	8116	10334
Rwork / Rfree	0.2303/0.3169	0.2515/0.3168	0.2336/0.2888
**No. atoms**			
Protein	1184	2310	1179
Ligand/ion	0		
Water	77	73	147
**R.m.s. deviations**			
Bond lengths (Å)	0.002	0.035	0.007
Bond angles (°)	0.714	2.703	1.008
Condition	0.2 M K2HPO4, 20 %PEG3350	1.4 M Sodium/Potassium Phosphate pH 8.2	0.05 M citric acid, 0.05 M BIS-TRIS propane pH 5.0, 16 % PEG 3350

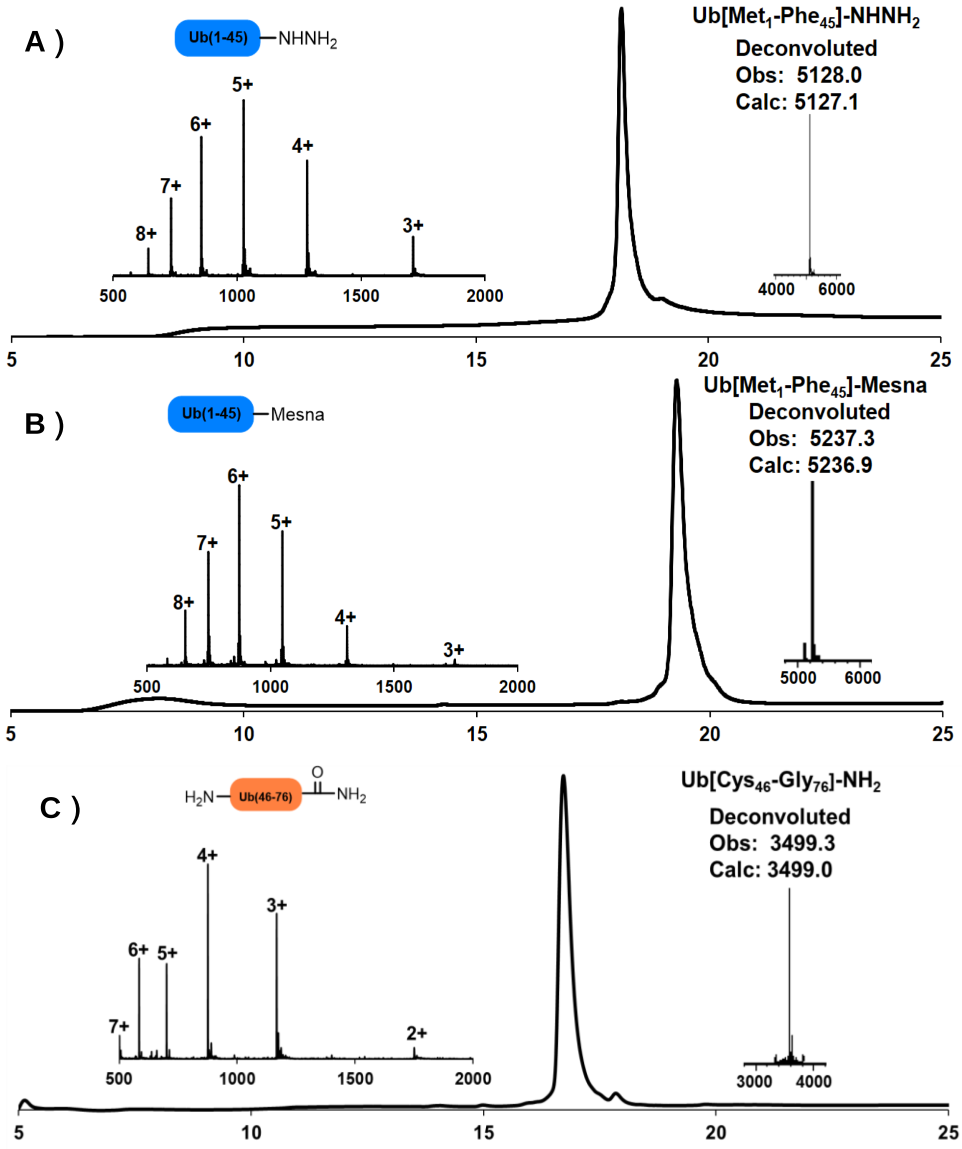

Figure S1 Characterization of segments 4 and 1 and the corresponding Mesna. A) Analytical HPLC chromatogram of isolated Ub[Met_1-Phe_{45}]-$NHNH_2$. ESI-MS spectrum (8+, 641.9 Da; 7+, 733.5 Da; 6+, 855.6 Da; 5+, 1026.5 Da; 4+, 1282.8 Da; and 3+, 1710.1 Da, observed mass = 5128.0 Da, calculated = 5127.1 Da). B) Analytical HPLC chromatogram of isolated Ub[Met_1-Phe_{45}]-Mesna. ESI-MS spectrum (8+, 655.7 Da; 7+, 749.2 Da; 6+, 874.0 Da; 5+, 1048.4 Da; 4+, 1310.3 Da; and 3+, 1746.8 Da, observed mass = 5237.3 Da, calculated = 5236.9 Da). C) Analytical HPLC chromatogram of isolated Ub[Cys_{46}-Gly_{76}]-NH_2. ESI-MS spectrum (7+, 501.0 Da; 6+, 584.3 Da; 5+, 700.9 Da; 4+, 875.9 Da; 3+, 1167.5 Da; and 2+, 1750.8 Da, observed mass = 3578.1 Da, calculated = 3579.0 Da). (Note: HPLC conditions: Welch 'XB-C18', a linear gradient of 20-60% acetonitrile (containing 0.1% TFA) in water (containing 0.1% TFA) over 30 min, $\lambda = 214$ nm).

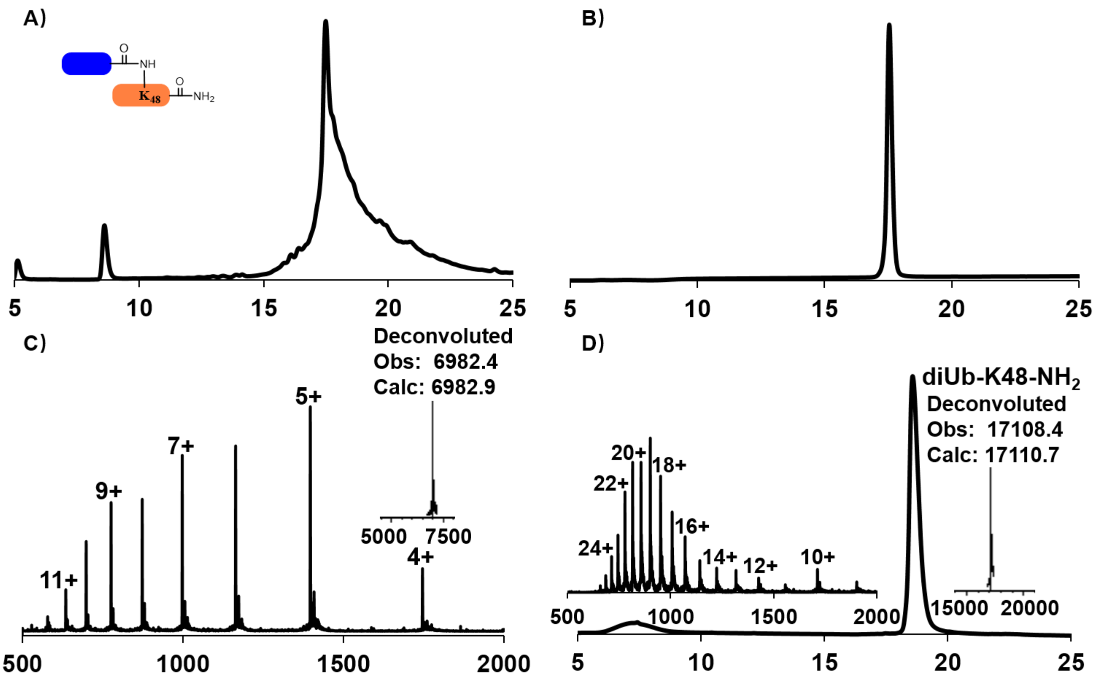

Figure S2 Synthesis and characterization of K48-diUb. A) Analytical HPLC chromatogram of crude Ub[Cys_{46}-(Cys_{46}-Gly_{76})Lys_{48}-Gly_{76}]-NH_2. B) Analytical HPLC chromatogram of isolated Ub[Cys_{46}-(Cys_{46}-Gly_{76})Lys_{48}-Gly_{76}]-NH_2. C) ESI-MS spectrum of isolated Ub[Cys_{46}-(Cys_{46}-Gly_{76})Lys_{48}-Gly_{76}]-NH_2. ESI-MS (11+, 635.7 Da; 9+, 776.8 Da; 7+, 998.4 Da; 5+, 1397.4 Da; and 4+, 1746.6 Da, observed mass = 6982.4 Da, calculated = 6982.9 Da). D) Analytical HPLC chromatogram of isolated K48-diUb. ESI-MS (24+, 713.8 Da; 22+, 778.6 Da; 20+, 856.5 Da; 18+, 951.5 Da; 16+, 1070.3 Da; 14+, 1223.1; 12+, 1426.6; and 10+, 1711.9, observed mass = 17108.4 Da, calculated = 17110.7 Da). (Note: HPLC conditions: Welch 'XB-C4', a linear gradient of 25-60% acetonitrile (containing 0.1% TFA) in water (containing 0.1% TFA) over 30 min, λ = 214 nm).

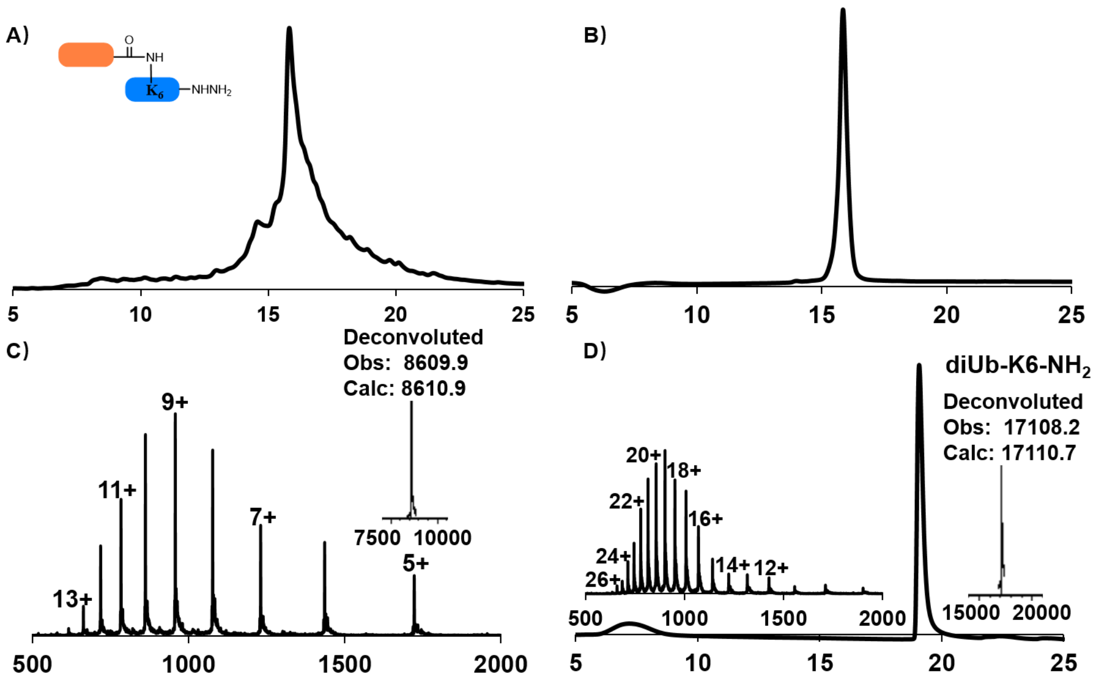

Figure S3 Synthesis and characterization of K6-diUb. A) Analytical HPLC chromatogram of crude Ub[Met_1-(Cys_{46}-Gly_{76})Lys_6-Phe_{45}]-$NHNH_2$. B) Analytical HPLC chromatogram of isolated Ub[Met_1-(Cys_{46}-Gly_{76})Lys_6-Phe_{45}]-$NHNH_2$. C) ESI-MS spectrum of isolated Ub[Met_1-(Cys_{46}-Gly_{76})Lys_6-Phe_{45}]-$NHNH_2$. ESI-MS spectrum (13+, 663.3 Da; 11+, 783.7 Da; 9+, 957.7 Da; 7+, 1231.0 Da; and 5+, 1723.0 Da, observed mass = 8609.9 Da, calculated = 8610.9 Da). D) Analytical HPLC chromatogram of isolated K6-diUb. ESI-MS spectrum (26+, 659.0 Da; 24+, 713.9 Da; 22+, 778.7 Da; 20+, 856.4 Da; 18+, 951.5 Da; 16+, 1070.3 Da; 14+, 1223.0 Da; and 12+, 1426.6 Da, observed mass = 17108.2 Da, calculated = 17110.7 Da). (Note: HPLC conditions: Welch 'XB-C4', a linear gradient of 25-60% acetonitrile (containing 0.1% TFA) in water (containing 0.1% TFA) over 30 min, λ = 214 nm).

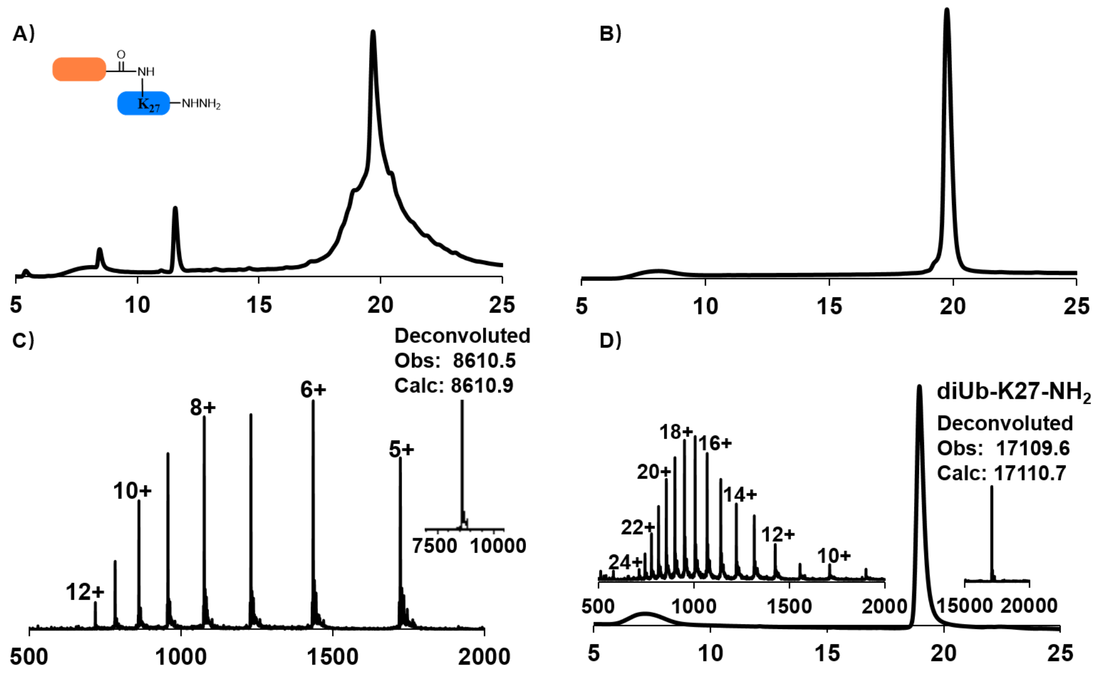

Figure S4 Synthesis and characterization of K27-diUb. A) Analytical HPLC chromatogram of crude Ub[Met_1-(Cys_{46}-Gly_{76})Lys_{27}-Phe_{45}]-$NHNH_2$. B) Analytical HPLC chromatogram of isolated Ub[Met_1-(Cys_{46}-Gly_{76})Lys_{27}-Phe_{45}]-$NHNH_2$. C) ESI-MS spectrum of isolated Ub[Met_1-(Cys_{46}-Gly_{76})Lys_{27}-Phe_{45}]-$NHNH_2$. ESI-MS spectrum (12+, 718.6 Da; 10+, 862.0 Da; 8+, 1077.3 Da; 6+, 1436.1 Da; and 5+, 1723.2 Da, observed mass = 8610.5 Da, calculated = 8610.9 Da). D) Analytical HPLC chromatogram of isolated K27-diUb. ESI-MS spectrum (24+, 713.9 Da; 22+, 778.7 Da; 20+, 856.5 Da; 18+, 951.5 Da; 16+, 1070.4 Da; 14+, 1223.1 Da; 12+, 1426.7 Da; and 10+, 1712.0 Da, observed mass = 17109.6 Da, calculated = 17110.7 Da). (Note: HPLC conditions: Welch 'XB-C4', a linear gradient of 25-60% acetonitrile (containing 0.1% TFA) in water (containing 0.1% TFA) over 30 min, λ = 214 nm).

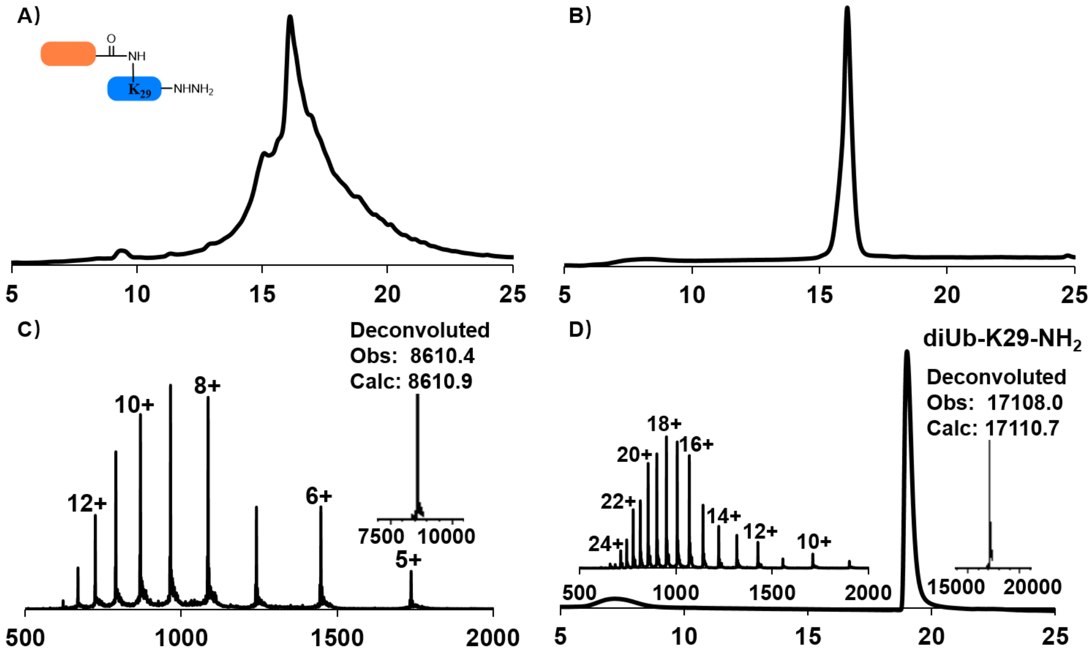

Figure S5 Synthesis and characterization of K29-diUb. A) Analytical HPLC chromatogram of crude Ub[Met_1-(Cys_{46}-Gly_{76})Lys_{29}-Phe_{45}]-$NHNH_2$. B) Analytical HPLC chromatogram of isolated Ub[Met_1-(Cys_{46}-Gly_{76})Lys_{29}-Phe_{45}]-$NHNH_2$. C) ESI-MS spectrum of isolated Ub[Met_1-(Cys_{46}-Gly_{76})Lys_{29}-Phe_{45}]-$NHNH_2$. ESI-MS spectrum (12+, 718.6 Da; 10+, 862.0 Da; 8+, 1077.3 Da; 6+, 1436.0 Da; and 5+, 1723.2 Da, observed mass = 8610.4 Da, calculated = 8610.9 Da). D) Analytical HPLC chromatogram of isolated K29-diUb. ESI-MS spectrum (24+, 713.8 Da; 22+, 778.7 Da; 20+, 856.4 Da; 18+, 951.4 Da; 16+, 1070.2 Da; 14+, 1223.0 Da; 12+, 1426.6 Da; and 10+, 1711.7 Da, observed mass = 17108.0 Da, calculated = 17110.7 Da). (Note: HPLC conditions: Welch 'XB-C18', a linear gradient of 25-60% acetonitrile (containing 0.1% TFA) in water (containing 0.1% TFA) over 30 min, λ = 214 nm).

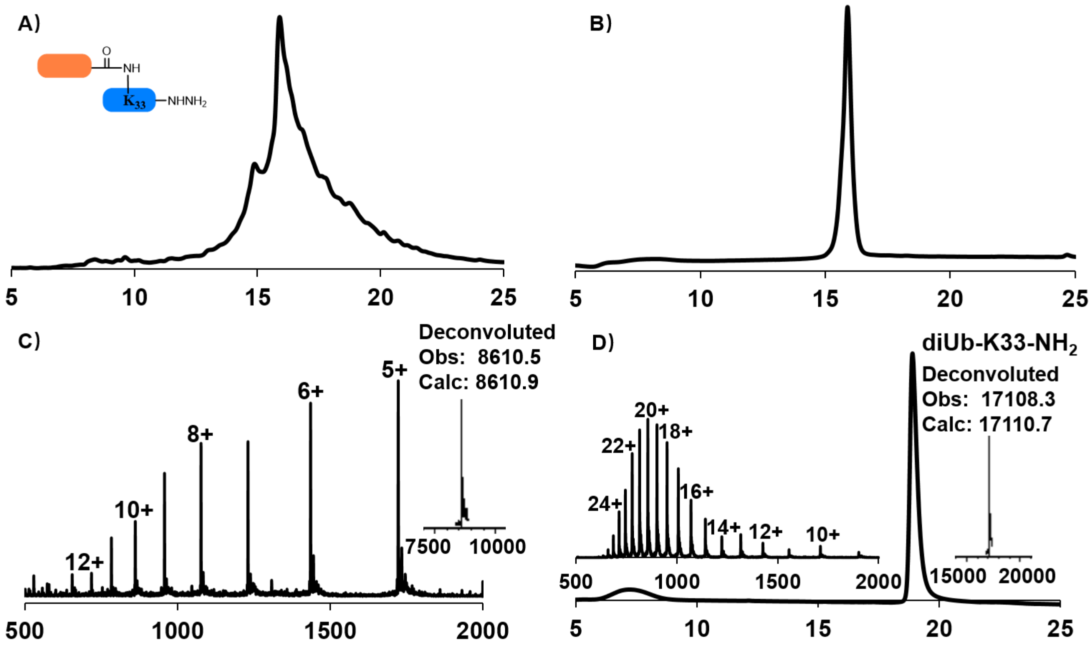

Figure S6 Synthesis and characterization of K33-diUb. A) Analytical HPLC chromatogram of crude Ub[Met_1-(Cys_{46}-Gly_{76})Lys_{33}-Phe_{45}]-$NHNH_2$. B) Analytical HPLC chromatogram of isolated Ub[Met_1-(Cys_{46}-Gly_{76})Lys_{33}-Phe_{45}]-$NHNH_2$. C) ESI-MS spectrum of isolated Ub[Met_1-(Cys_{46}-Gly_{76})Lys_{33}-Phe_{45}]-$NHNH_2$. ESI-MS spectrum (12+, 718.6 Da; 10+, 862.0 Da; 8+, 1077.2 Da; 6+, 1436.1 Da; and 5+, 1723.1 Da, observed mass = 8610.5 Da, calculated = 8610.9 Da). D) Analytical HPLC chromatogram of isolated K33-diUb. ESI-MS spectrum (24+, 713.8 Da; 22+, 778.6 Da; 20+, 856.3 Da; 18+, 951.4 Da; 16+, 1070.2 Da; 14+, 1222.9 Da; 12+, 1426.5 Da; and 10+, 1711.7 Da, observed mass = 17108.3 Da, calculated = 17110.7 Da). (Note: HPLC conditions: Welch 'XB-C4', a linear gradient of 25-60% acetonitrile (containing 0.1% TFA) in water (containing 0.1% TFA) over 30 min, λ = 214 nm).

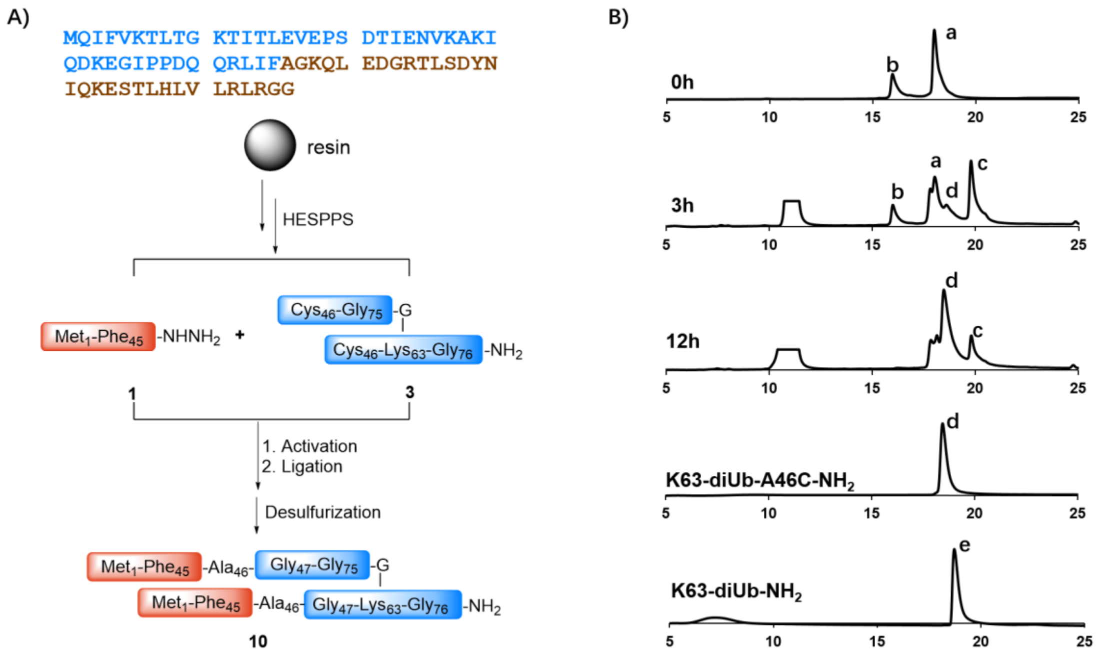

Figure S7 Synthesis of diUb-K63. A) Synthetic route. B) Analytical HPLC chromatogram of the native chemical ligation reaction. (peak a, Ub[Met_1-Phe_{45}]-Mesna; peak b, Ub[Cys_{46}-(Cys_{46}-Gly_{76})Lys_{63}-Gly_{76}]-NH_2; peak c, Ub[Met_1-Phe_{45}]-MPAA; peak d, K63-diUb-A46C-NH_2; peak e, K63-diUb-NH_2). HPLC conditions: 'grace-C8', a linear gradient of 20-60% acetonitrile (containing 0.1% TFA) in water (containing 0.1% TFA) over 30 min, $\lambda = 214$ nm.

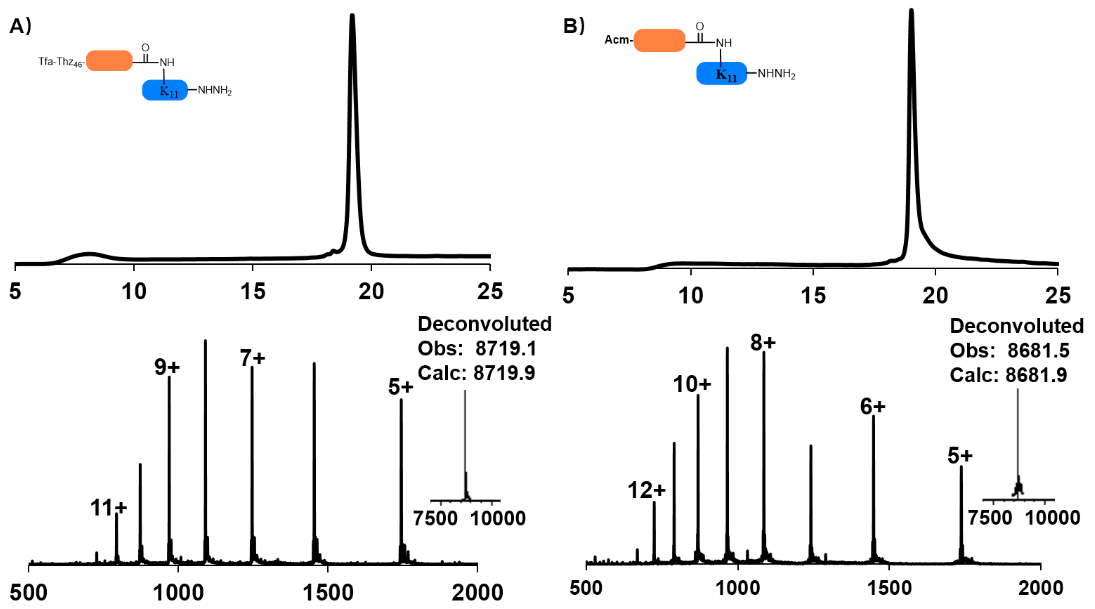

Figure S8 Synthesis and characterization of key building blocks 11 and 11a. A) Analytical HPLC chromatogram of crude and isolated Ub[Met$_1$-(Tfa-Thz$_{46}$-Gly$_{76}$)Lys$_{11}$-Phe$_{45}$]-NHNH$_2$. ESI-MS spectrum (11+, 793.6 Da; 9+, 969.7 Da; 7+, 1246.5 Da; 5+, 1744.8 Da, observed mass = 8719.1 Da, calculated = 8719.9 Da). B) Analytical HPLC chromatogram of isolated Ub[Met$_1$-(Acm-Cys$_{46}$-Gly$_{76}$)Lys$_{11}$-Phe$_{45}$]-NHNH$_2$. ESI-MS spectrum (12+, 724.5 Da; 10+, 869.2 Da; 8+, 1086.2 Da; 6+, 1447.9 Da; and 5+, 1737.3 Da, observed mass = 8681.5 Da, calculated = 8681.9 Da). (Note: HPLC conditions: Welch 'XB-C4', a linear gradient of 20-60% acetonitrile (containing 0.1% TFA) in water (containing 0.1% TFA) over 30 min, λ = 214 nm).

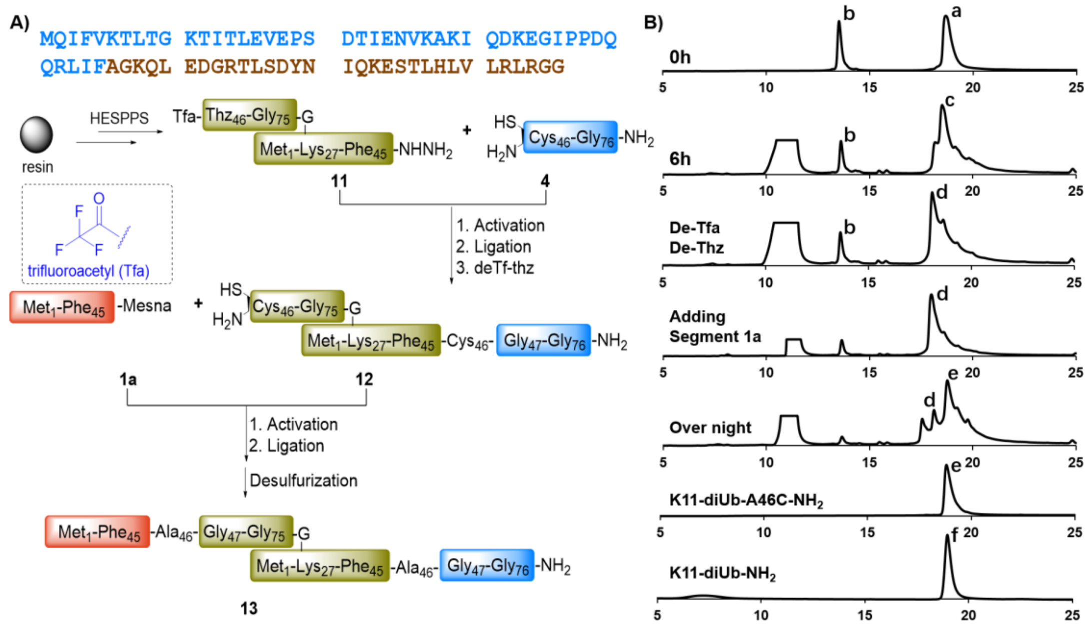

Figure S9 Synthesis of K11-diUb. A) Synthetic route. B) Analytical HPLC chromatogram of the native chemical ligation reaction. (peak a, Ub[Met_1-(Tfa-Thz_{46}-Gly_{76})Lys_{11}-Phe_{45}]-$NHNH_2$; peak b, Ub[Cys_{46}-Gly_{76}]-NH_2; peak c, Ub[Met_1-(Tfa-Thz_{46}-Gly_{76})Lys_{11}-Cys_{46}-Gly_{76}]-NH_2; peak d, Ub[Met_1-(Cys_{46}-Gly_{76})Lys_{11}-Cys_{46}-Gly_{76}]-NH_2; peak e, K11-diUb-(A46C)-NH_2; peak f, K11-diUb-NH_2). HPLC conditions: 'grace-C8', a linear gradient of 20-60% acetonitrile (containing 0.1% TFA) in water (containing 0.1% TFA) over 30 min, λ = 214 nm.

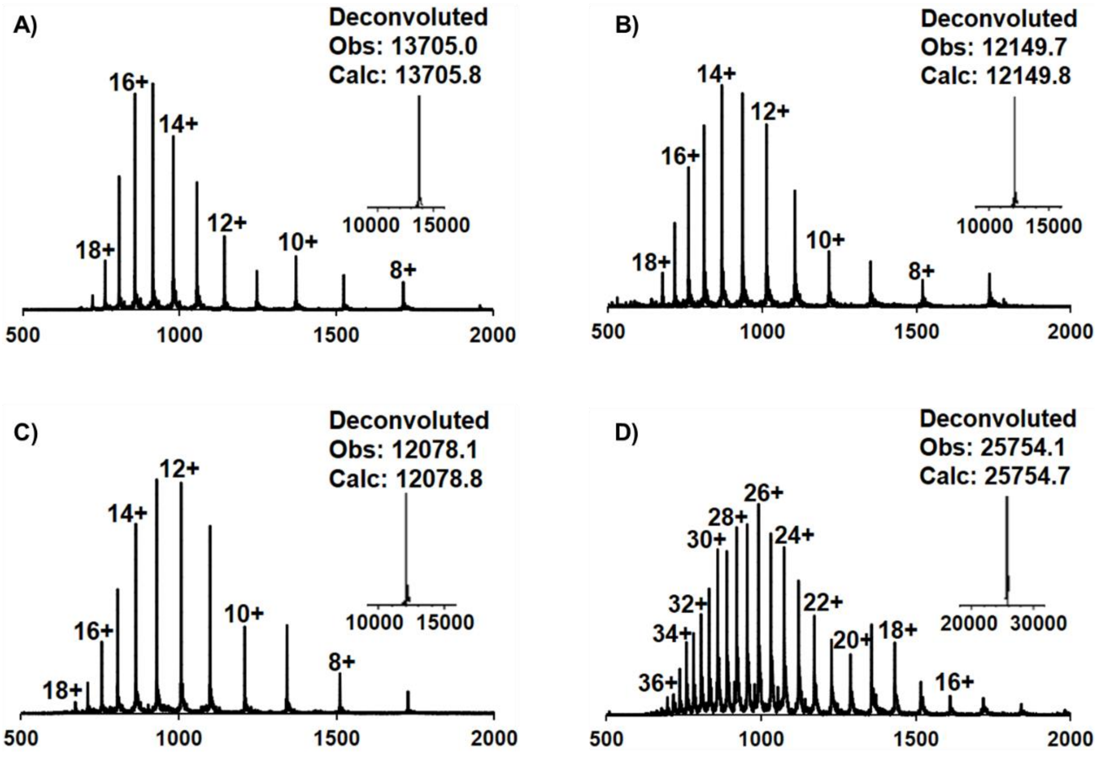

Figure S10 Synthesis of K33/K11-triUb. A) ESI-MS spectrum of isolated Ub[Met_1-(Met_1-Cys_{46}-Gly_{76})Lys_{33}-Phe_{45}]-$NHNH_2$ (18+, 762.5 Da; 16+, 857.6 Da; 14+, 979.9 Da; 12+, 1143.2 Da; 10+, 1371.5 Da; and 8+, 1714.0 Da, observed mass = 13705.0 Da, calculated = 13705.8 Da). B) ESI-MS spectrum of isolated Ub[Met_1-(Acm-Cys_{46}-Gly_{76})Lys_{11}-Cys_{46}-Gly_{76}]-NH_2 (18+, 676.0 Da; 16+, 760.4 Da; 14+, 868.9 Da; 12+, 1013.5 Da; 10+, 1216.1 Da; and 8+, 1519.7 Da, observed mass = 12149.7 Da, calculated = 12149.8 Da). C) ESI-MS spectrum of isolated Ub[Met_1-(Cys_{46}-Gly_{76}) Lys_{11}-Cys_{46}-Gly_{76}]-NH_2 (16+, 755.9 Da; 14+, 863.7 Da; 12+, 1007.5 Da; 10+, 1208.8 Da; and 8+, 1510.8 Da, observed mass = 12078.1 Da, calculated = 12078.8 Da). D) ESI-MS sepctrum of isolated triUb-K33/K11-A46C-NH_2 (34+, 758.5 Da; 32+, 805.8 Da; 30+, 859.5 Da; 28+, 920.8 Da; 26+, 991.6 Da; 24+, 1074.2 Da; 22+, 1171.7 Da; 20+, 1288.7 Da; 18+, 1431.8 Da; and 16+, 1610.7 Da, observed mass = 25754.1 Da, calculated = 25754.7 Da).

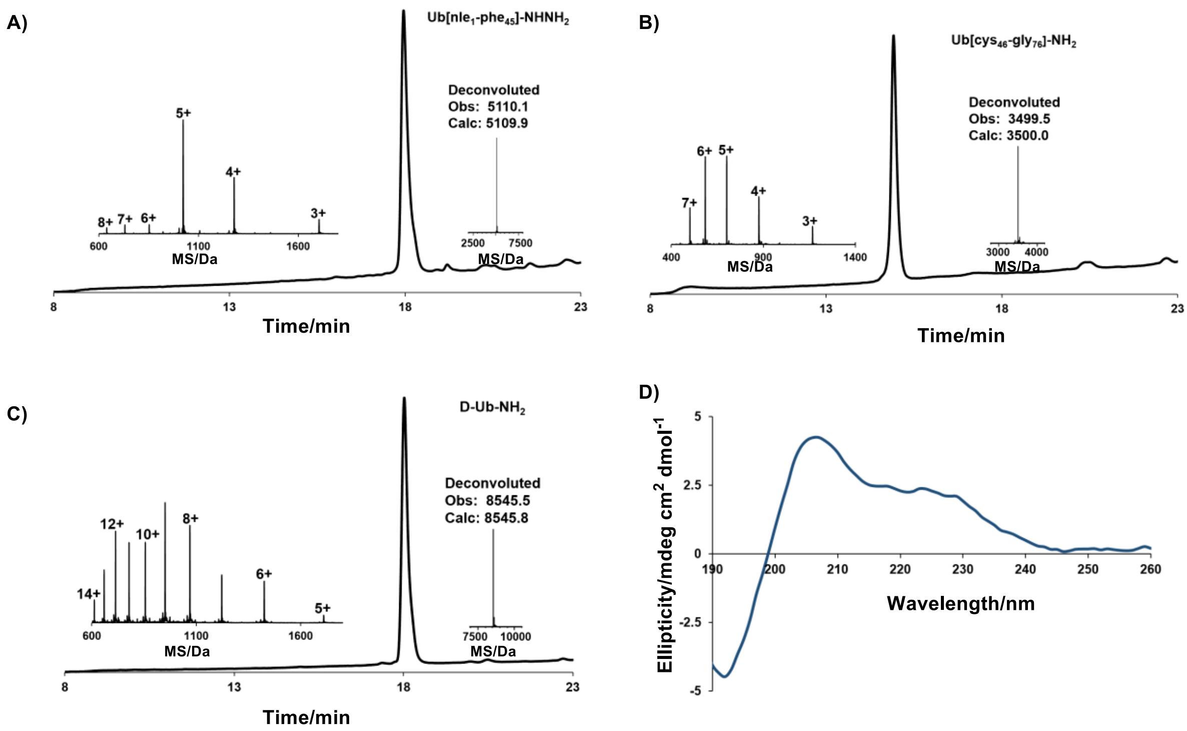

Figure S11 Synthesis of D-Ub-NH$_2$. A) Analytical HPLC chromatogram of crude Ub[Nle$_1$-Phe$_{45}$]-NHNH$_2$. Inset: Deconvoluted ESI-MS spectrum (8+, 641.9 Da; 7+, 733.5 Da; 6+, 855.6 Da; 5+, 1026.5 Da; 4+, 1282.9 Da; and 3+, 1710.3 Da, observed mass = 5110.1 Da, calculated = 5109.9 Da, average isotopes). B) Analytical HPLC chromatogram of isolated Ub[Cys$_{46}$-Gly$_{76}$]-NH$_2$. Inset: Deconvoluted ESI-MS spectrum (7+, 501.0 Da; 6+, 584.3 Da; 5+, 700.9 Da; 4+, 875.9 Da; 3+, 1167.5 Da, observed mass = 3499.5 Da, calculated =3500.0 Da, average isotopes). C) Analytical HPLC chromatogram of isolated d-Ub-NH$_2$. ESI-MS spectrum (14+, 612.9 Da; 12+, 714.7 Da; 10+, 857.4 Da; 8+, 1071.5 Da; 6+, 1428.3 Da; and 5+, 1713.8 Da, observed mass = 8545.5 Da, calculated = 8545.8 Da). D) CD spectra of D-mono-Ub. (Note: HPLC conditions: Welch 'XB-C4', a linear gradient of 25-60% acetonitrile (containing 0.1% TFA) in water (containing 0.1% TFA) over 30 min, λ = 214 nm).
